# Supplementary figures and images for: Characteristic and quantifiable COVID-19-like abnormalities in CT- and PET/CT-imaged lungs of SARS-CoV-2-infected crab-eating macaques (Macaca fascicularis)
Source: bioRxiv. 2020 May 14:2020.05.14.096727. Preprint. [Version 1] doi: 10.1101/2020.05.14.096727 (PMC7241101; doi:10.1101/2020.05.14.096727)

Supplementary Figure 1

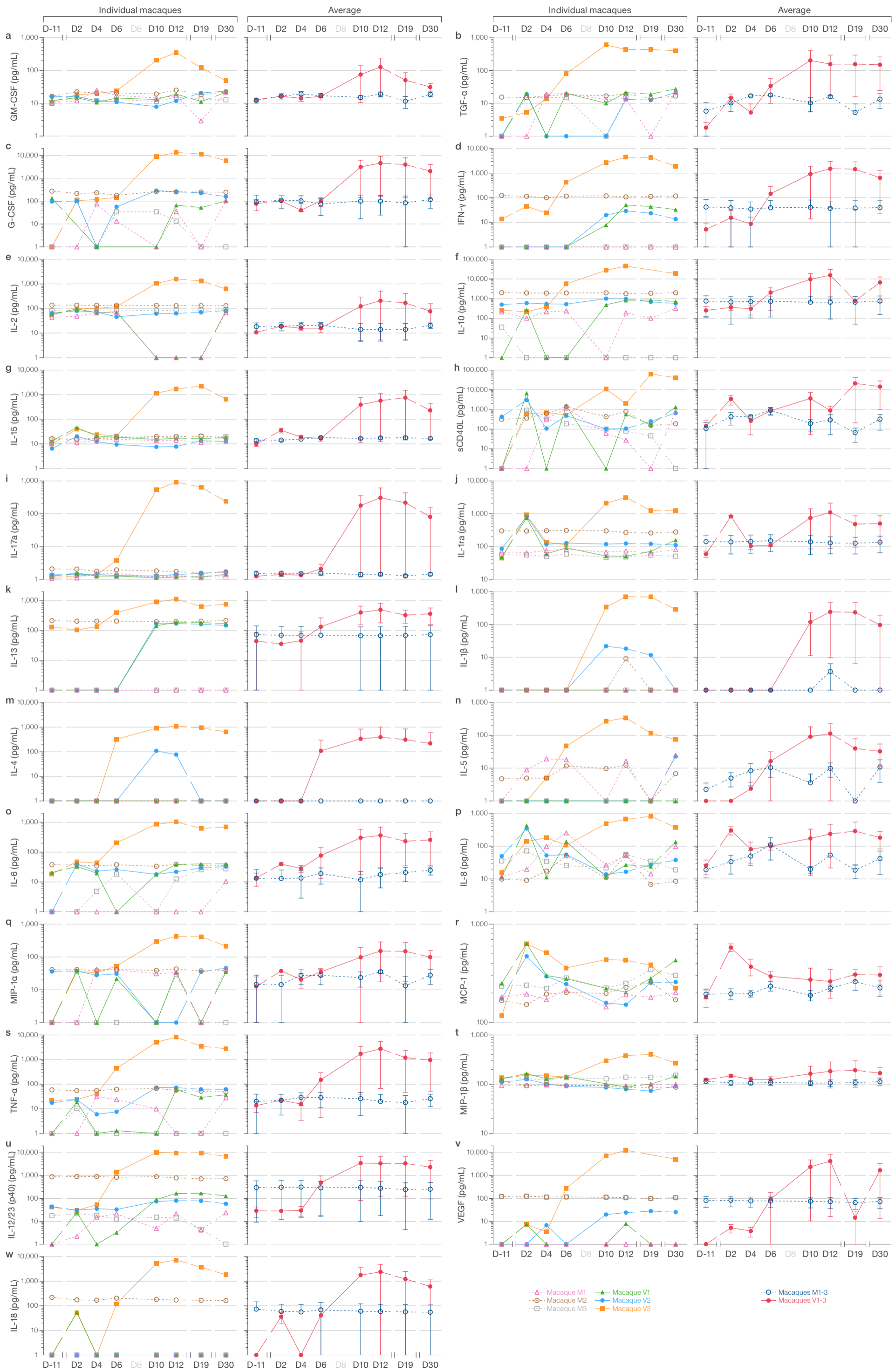

Supplement: Supplement 1 — Supplementary Figure 1 | Cytokines. Cytokien concentration changes measured for individual macaques and averaged for mock group (M) and virus group (V) macaques. Data are represented on logarithmic scales. For graphic representation, values of “0” or “not detected” (below the level of assay sensitivity) were automatically assigned a value = 1. [file media-1.pdf]

Supplementary Figure 2

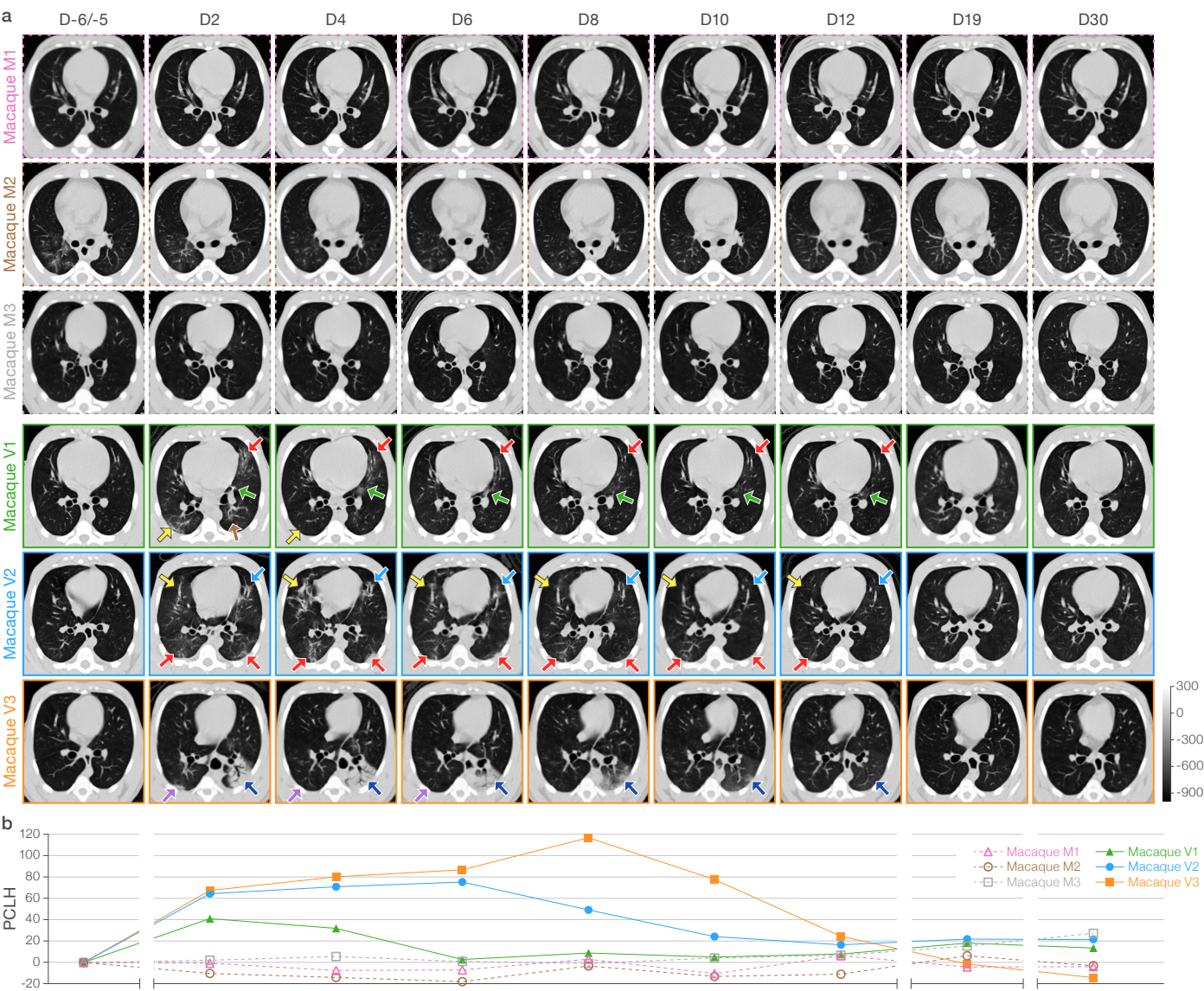

Supplement: Supplement 2 — Supplementary Figure 2 | Qualitative and quantitative computed tomography (CT) analysis of macaque lungs. a) Representative axial CT images in three SARS-CoV-2-infected (V) macaques and mock-inoculated (M) macaques for all indicated study days (D). The grey scale represents radiodensity in Hounsfield units (HU). Selected CT images (axial, sagittal, and coronal views) from the SARS-CoV-2 infected macaques with detailed radiological descriptions are shown in Supplementary Figure 3. Colored arrows in Figure 4 and this figure represent regions of interest that are further detailed in figure legends for Supplementary Figure 3. b) Percent change in lung hyperdensity (PCLH) measured over time in the same macaques shown in Figure 4, including also here the mock-infected individual macaques and all study days. [file media-2.pdf]

Supplementary Figure 4

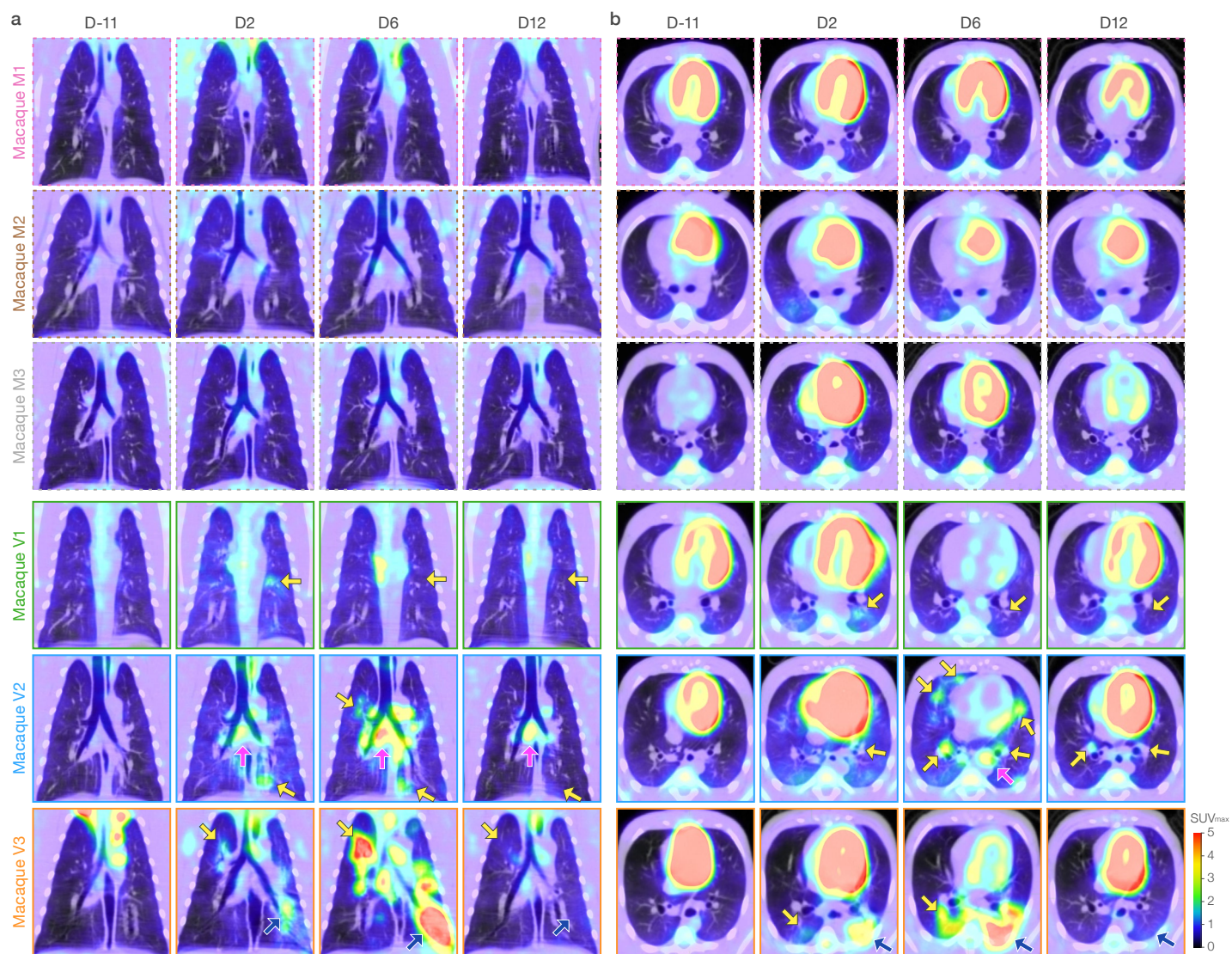

Supplement: Supplement 4 — Supplementary Figure 4 | Qualitative positron emission tomography (PET) and PET/CT analysis of macaque lungs. Representative coronal (left panels) and axial (right panels) 2-deoxy-2-[18F]-fluoro-D-glucose (FDG) PET/CT images for each indicated study day (D). SUVmax, mean FDG maximum standardized uptake values. Selected areas of increased FDG uptake are highlighted in the lung parenchyma (yellow arrows) and regional lymph nodes (pink arrows). Selected merged PET/CT images (axial, sagittal, and coronal views) with detailed radiological descriptions of these areas of interest are shown in Supplementary Figure 5. [file media-4.pdf]
