# Supplementary material for: Characteristic and quantifiable COVID-19-like abnormalities in CT- and PET/CT-imaged lungs of SARS-CoV-2-infected crab-eating macaques (Macaca fascicularis)
Source: bioRxiv. 2020 May 14:2020.05.14.096727. Preprint. [Version 1] doi: 10.1101/2020.05.14.096727 (PMC7241101; doi:10.1101/2020.05.14.096727)
Supplement: Supplement 6 — Supplementary Table 1 | Crab-eating macaque (Macaca fascicularis Raffles, 1821) information. [file media-6.pdf]

Supplementary Table 1 | Crab-eating macaque (*Macaca fascicularis* Raffles, 1821) information

**Group**	**Macaque ID**	**Age**	**Weight at baseline (kg)**	**Sex**	**Inoculum**
Mock (M)	M1	4 years 4 months	3.29	F	DMEM + 2% heat-inactivated FBS
	M2	3 years 11 months	3.17	F	
	M3	3 years 9 months	4.87	M	
Virus (V)	V1	4 years 3 months	4.34	M	DMEM + 2% heat-inactivated FBS + $3.65x10^6$ pfu SARS-CoV-2
	V2	4 years	4.62	M	
	V3	4 years 4 months	3.17	F	
