# Supplementary material for: Characteristic and quantifiable COVID-19-like abnormalities in CT- and PET/CT-imaged lungs of SARS-CoV-2-infected crab-eating macaques (Macaca fascicularis)
Source: bioRxiv. 2020 May 14:2020.05.14.096727. Preprint. [Version 1] doi: 10.1101/2020.05.14.096727 (PMC7241101; doi:10.1101/2020.05.14.096727)
Supplement: Supplement 9 — Supplementary Table 4 | Macaque clinical scoring guide. [file media-9.pdf]

Clinical Scoring

Cage-Side Assessment			
**Parameter**	**Description**	**Score**	
Responsiveness and recumbency	Normal—bright, alert, responsive	0	
	Mild—slightly depressed, acts disinterested when personnel in room, lying down in cage but gets up when approached	2	
	Moderate/obtunded—non-responsive, very disinterested in personnel, hunched or lying down, will get up when prodded, pinched, or similarly stimulated	4	
	Severe/comatose—lying down completely unresponsive to stimuli	6	
Discharges	Nasal or ocular	2	
	Nasal and ocular	4	
Skin	Rash	1	
Respiration, dyspnea, and cough	Normal—no apparent changes in breathing, 30–54 breath per minute (BPM), and no cough	0	
	Mild—slightly increased effort breathing, 55–65 BPM, or mild cough	2	
	Moderate—obvious difficulty breathing, 66–80 BPM, or apparent cough	4	
	Severe—respirations labored, open mouth breathing, abdominal breathing, >80 BPM, cyanosis, or hemoptysis	6	
Food consumption	100% of biscuits	0	
	10–25% of biscuits remaining	1	
	25–50% of biscuits remaining	2	
	>50% of biscuits remaining	3	
Fecal consistency	Normal	0	
	Soft	1	
	Fluid	2	
	Fluid and profuse amount	3	
		**Total**	

Notes *(any observed sneezing, vomit, conjunctival erythema, or other abnormalities*)

**Physical Examination Under Anesthesia**			
**Parameter**	**Description**	**Score**	
Rectal temperature *(taken immediately after sedation)*	Normal (37.0–38.9°C)	0	
	Low-grade fever (39–39.5°C)	2	
	Fever (>39.5°C)	4	
Heart rate	Normal (up to 19 BPM over baseline, ***/***)	0	
	Mild tachycardia (20–39 BPM over baseline),	1	
	Moderate tachycardia (40–69 BPM over baseline)	2	
	Severe tachycardia (>70 BPM over baseline)	3	
Respiratory rate	Normal—30–54 BPM	0	
	Mild tachypnea—55–65 BPM	2	
	Moderate tachypnea—66–80 BPM	4	
	Severe tachypnea—>80 BPM	6	
SpO_2	Normal (95–100%)	0	
	Mildly decreased (90–94%)	1	
	Moderately decreased (87–89)	2	
	Severely decreased (<87)	3	
Body weight	Normal (0–3% loss)	0	
	Mild (4–9% loss)	1	
	Moderate (10–16% loss)	2	
	Severe (>16% loss)	3	
Dehydration	Normal skin turgor, moist mucous membranes	0	
	Skin tenting or dry mucous membranes	1	
	Skin tenting and dry mucous membranes	2	
	Skin tenting, dry mucous membranes, and sunken eyes	3	
		**Total**	

Notes *(auscultation findings if applicable, conjunctival erythema, palpable masses, or any other abnormalities)*

The sheets above were modified from a previous nonhuman primate influenza A virus study to include clinical signs relevant to COVID-19 and respiratory rates for crab-eating macaques (1-3). Physical examinations were performed whenever an animal was anesthetized.

References

1. D. S. Chertow *et al.*, Influenza A and methicillin-resistant Staphylococcus aureus co-infection in rhesus macaques - A model of severe pneumonia. *Antiviral Res* **129**, 120-129 (2016).
2. I. D. Bolton, in *The nonhuman primate in nonclinical drug development and safety assessment,* J. Bluemel, S. Korte, E. Schenck, G. F. Weinbauer, Eds. (Academic Press, San Diego, CA, USA, 2015), pp. 67-86.
3. C. Huang *et al.*, Clinical features of patients infected with 2019 novel coronavirus in Wuhan, China. *Lancet* **395**, 497-506 (2020).
